# *It provides families with other avenues for treatment when there are no other options* Surgeons' perspectives of being part of a precision medicine trial for poor prognosis paediatric cancer patients: A short report

**DOI:** 10.1002/cam4.7209

**Published:** 2024-05-08

**Authors:** Rebecca Daly, Kate Hetherington, Bethany R. Wadling, Chris Jacobs, Jonathan Karpelowsky, Claire E. Wakefield

**Affiliations:** ^1^ Discipline of Paediatrics and Child Health, School of Clinical Medicine UNSW Medicine & Health, UNSW Sydney New South Wales Australia; ^2^ Behavioural Sciences Unit, Kids Cancer Centre Sydney Children's Hospital Randwick New South Wales Australia; ^3^ Graduate School of Health University of Technology Sydney Sydney New South Wales Australia; ^4^ Department of Paediatric Surgery Children's Hospital at Westmead Westmead New South Wales Australia; ^5^ Children's Cancer Research Unit Kids Research Institute, Children's Hospital at Westmead Westmead New South Wales Australia; ^6^ Division of Child and Adolescent Health University of Sydney Camperdown New South Wales Australia

**Keywords:** cancer, precision medicine, surgeon

## Abstract

**Objective:**

Precision medicine is transforming cancer treatment, yet the perspectives of surgeons who often play a critical role in the delivery of precision medicine remain understudied.

**Methods:**

We conducted semi‐structured interviews with 13 surgeons involved in a precision medicine trial for children with poor prognosis cancer. We explored knowledge of genetics, confidence with somatic and germline results, ratings of benefit to stakeholders and willingness to undertake surgical procedures.

**Results:**

Surgeons generally had positive attitudes towards precision medicine but expressed concerns about families' unrealistic expectations, mixed opinions on the benefits and the use of research‐only biopsies. Most surgeons rated their genetics knowledge as ‘good’ (69%) and felt ‘very confident’ in identifying genetic specialists (66%), but ‘not confident’ (66.6%) in making treatment recommendations. Surgeons' willingness to undertake a procedure was influenced by potential patient benefit.

**Conclusions:**

Our findings support the need for more workforce and training support for surgeons to fully engage with precision medicine.

## 1 INTRODUCTION

Over 80% of children diagnosed with cancer are cured using traditional treatment methods such as chemotherapy, radiation and surgery.^1^ However, there remains a subset of children with poor prognosis cancers for whom novel treatment options offer hope through precision medicine.^2^ Precision medicine focuses on matching the most effective treatment to each individual patient based on their cancers and individual genetic profile.^3^ This approach aims to maximise survival, minimise side effects from traditional treatments and avoid unnecessary treatments that are likely to be ineffective.^4^

Surgeons play a fundamental role in cancer treatment particularly paediatric sarcoma treatment, where surgery is a major modality.^5^ Participation in precision medicine programmes often requires submission of tissue for molecular assessment with surgeons called upon to biopsy newly diagnosed, refractory or relapsed tumours more frequently.^2^

Precision medicine will impact surgical care from preoperative counselling to decision‐making^2^ requiring surgeons to have increased knowledge of genetics and its interaction with modern surgical practice.^6^

Exploring surgeons' perspectives as precision medicine is integrated into cancer care is important to identify potential barriers to implementation and to gain an understanding of surgeons' needs and concerns as they navigate the resulting changes.^7^ Surgeons' perspectives of integrating precision medicine into cancer care are understudied. To address this gap, we recruited surgeons to answer the following research questions:

- What are surgeons' experiences of and attitudes towards precision medicine?
- What are surgeons' experiences of collecting samples and biopsies in a precision medicine trial?
- How confident are surgeons in their knowledge and understanding of genetic concepts?

## 2 METHOD

### 2.1 PRISM and PRISM‐impact

The PRecISion Medicine for Children with Cancer (PRISM) study is a national precision medicine trial for paediatric and adolescent patients (≤21 years) with poor prognosis malignancies (expected survival <30%) embedded in the Zero Childhood Cancer Program (Zero)^8^ (Australian and New Zealand Clinical Trials Registry (NCT03336931)). We previously published data from PRISM‐Impact which includes an overview of the roles of various health professionals (including surgeons) in the PRISM trial.^7^ Surgeons were involved in obtaining tissue samples required for PRISM enrolment. Surgeons were not routinely included in the PRISM MTB. PRISM‐Impact is a mixed methods prospective psychosocial sub‐study that aims to better understand family and health professionals' experiences of precision medicine. PRISM and PRISM‐Impact were conducted in accordance with the Declaration of Helsinki and received Institutional Board Approval (17/02/15/4.06; HREC/17/HNE/29).

### 2.2 Procedure

We invited surgeons involved in the PRISM trial to participate in a semi‐structured telephone interview during its second year (February–July 2020). Potential participants were identified using the PRISM trial clinical database and sent invitations via a personalised email from a surgical lead (JW). Surgeons opted in via email reply. Two trained researchers (RD/NH) conducted the interviews which lasted 26:54 min on average (range 16:11–39:34). Interviews were audio‐recorded and transcribed verbatim.

### 2.3 Interview

The interview guide was developed by an expert multidisciplinary panel. Topics included hopes/expectations of precision medicine, experiences with PRISM, attitudes towards genetic testing and decision‐making regarding collecting biopsies. We collected an indication (as a percentage) of surgeons' willingness to undertake surgery in four scenarios with varying risk (from low–high risk) and benefit (from minimal/no benefit to current patient but potential benefit for future patients—high benefit) and asked surgeons to rank in order who they believed benefited most from PRISM so far (patients, parent/family members, future patients, doctors, scientists and optional ‘other’ with a free text response). We verbally administered and recorded ratings of surgeons' confidence (4‐point Likert scale adapted from Ref. [9]) and perceived knowledge of genetic concepts (adapted from Refs [10] and [11]).

### 2.4 Data analysis

We adopted a convergent parallel mixed‐method design^12^ to cross‐validate and corroborate findings from qualitative and quantitative data.^13^ For quantitative data, we used Statistical Package for the Social Sciences (SPSS version 28.01; IBM, USA) to conduct descriptive analyses of demographic information and Likert scale data. Qualitative data were analysed using an inductive thematic approach.^14^ Two coders (BW/RD) familiarised themselves with the data and independently coded one transcript to develop the initial coding framework. The framework was revised and further developed via discussion with two researchers (KH/CJ). Interviews were coded line by line, reviewed and revised, and any disagreements were discussed until consensus was reached.

## 3 RESULTS

### 3.1 Participants

We invited 21 surgeons, of whom 17 opted in (80.9% response rate) with 13 participants completing an interview (76.4% participation rate). Participants were 69% male, on average 49 (±7.6) years old (range 40–69). Table 1 summarises their demographic characteristics.

**TABLE 1:** Participant demographics.

Characteristic	Participants (*n* = 13)
Site, *n* (%)	
Sydney Children's Hospital	2 (15.4)
Royal Children's Hospital, Melbourne	3 (23.1)
Perth Children's Hospital	1 (7.7)
Queensland Children's Hospital	3 (23.1)
The Children's Hospital, Westmead	1 (7.7)
Monash Children's Hospital, Melbourne	3 (23.1)
Age (years), Mean (*SD*), Range	49 (±7.6), 40–69
Gender, *n* (%)	
Male	9 (69%)
Percentage of their time dedicated to research, mean (SD), range	8.71 (7.6), 2–30
Percentage of their time dedicated to oncology, mean (SD), range	28.5 (21.8), 5–80
Formal genetics training, *n* (%)	
No formal genetics training	6 (46)
Medical school only	2 (31)
Subsequent genetics training	2 (15)
Missing	3 (8)

Abbreviations: *n*, number; SD, standard deviation.

### 3.2 Attitudes towards precision medicine

A key theme from surgeon's qualitative data was ‘benefits of precision medicine’. Surgeons expressed optimism regarding PRISM, citing its potential to offer hope for targeted, less invasive treatments with fewer side effects. ‘*I suspect [because of PRISM] we will be doing less and less major tumour resections*’ (*ID 100*). Surgeons felt offering the trial to families of a child with a poor prognosis provided reassurance to families and clinical team that all options had been exhausted. ‘*PRISM provides families with other avenues for [their child's] treatment when there are no other options*’ (*ID 087*). Surgeons expressed concern that families may hold unrealistic expectations regarding potential benefits. ‘*I think it's seen as a last cure hope rather than as a research study*’ (*ID 022*). PRISM reports were deemed valuable by surgeons for enhancing knowledge guiding surgical decision‐making and for the treatment of future patients. Table 2 illustrates our qualitative findings with additional representative quotes. Quantitatively, surgeons held differing views regarding who benefited most and least from PRISM with an equal number endorsing patients (*n* = 4), future patients (*n* = 4) and scientists (*n* = 4) as benefitting most. The remaining participant rated doctors as benefiting most (*n* = 1). Future patients were most frequently endorsed as benefitting least (*n* = 4), followed by doctors and scientists (*n* = 3 each).

**TABLE 2:** Themes with representative quotes.

Theme	Illustrative quotes
Benefits of precision medicine	*‘PRISM is able to inform families that their child has a targetable mutation in a tumour’* (ID 084) *‘For families it provides an option with potential benefit for their child, but also for other children’* (ID 091). *‘[PRISM] gives extra treatment options when the options have been exhausted’* (ID 104) *‘I don't think it actually helps the patient's family right now, it probably helps future patients I guess is probably what I would say’* (ID 094) ‘*Something like PRISM, which so far to my understanding, it has a pretty good track record, lots of good results, lots of people sending tissue from around the place, lots of information being gained with a direct potential to benefit patients, I think that's different to some of the more peripheral studies that are going on, yes okay its super interesting, but who's it actually going to help? Particularly in the fledgling stages of a study*’. (090)
Perspectives and experiences of PRISM biopsies	*‘If the patient didn't require a procedure for their own treatment, given this is paediatrics they would all require an anaesthetic as well. I would say that I would not subject a child to something that would not benefit them at all’*. (ID 087) *‘PRISM requirements of fresh not frozen tissue impact planning for which surgeries take place when’* (ID 084) *‘In many cases for PRISM, possibly we would need to possibly take more invasive biopsies depending on how big the risk for that is would depend on whether we proceed’* (ID 022) *‘I would not do it if they're not going to benefit from it. You know, if it's just for research, I would not be doing just a biopsy, it has to lead to some sort of management of diagnostic yield because surgery is not without risk’* (ID 094) *‘If we were taking tissue for research that was regarded as futile, I wouldn't participate’* (ID 091)
Knowledge of and confidence with genetics	*‘In India for instance they [surgeons] do a lot of genomic testing themselves. In Australia, no, we don't know enough about it’*. (ID 100) *‘I think the surgeons should be aware of the tumours which have genetic susceptibility and be able to refer those patients to genetic services. So, we do that regularly so if we have a tumour or a genetic condition in our family history, we refer them patients to genetic services for further investigations and care’* (ID 088) *‘Unless surgeons are greatly upskilled, they should not be distributing genomic information to families’* (ID 093)

### 3.3 Perspectives and experiences of PRISM biopsies

Surgeons were most willing to perform surgeries with low risk and high potential benefit (96.6%), but less willing to undertake surgeries with high risk and high benefit (75.3%), low risk and minimal/no benefit (48%), or high risk and minimal/no benefit (16.9%) to the patient. All surgeons felt they had the capacity to refuse to perform a biopsy if they felt the risk outweighed the benefits. Qualitatively, surgeons shared concerns about obtaining biopsies from children solely for research purposes; ‘*I don't want to put the child at risk for the sake of treatment which is still I believe you know partly experimental not guaranteed success*’ (*ID 089*). Surgeons expressed difficulties obtaining sufficient biopsies with some expressing a willingness to perform additional or larger biopsies if they felt the risk was acceptable. ‘*Taking larger biopsies for PRISM is valid so long as it does not increase the risk for the child*’ (*ID 088*). Surgeons described the need for logistical considerations in providing fresh tissue for PRISM, which at times changed surgery scheduling. Most were willing to accommodate but highlighted potential financial impacts on the hospital system arising from these requirements such as extra time in theatre, use of more instruments and evening/weekend procedures.

### 3.4 Knowledge of and confidence with genetics

**FIGURE 1:**
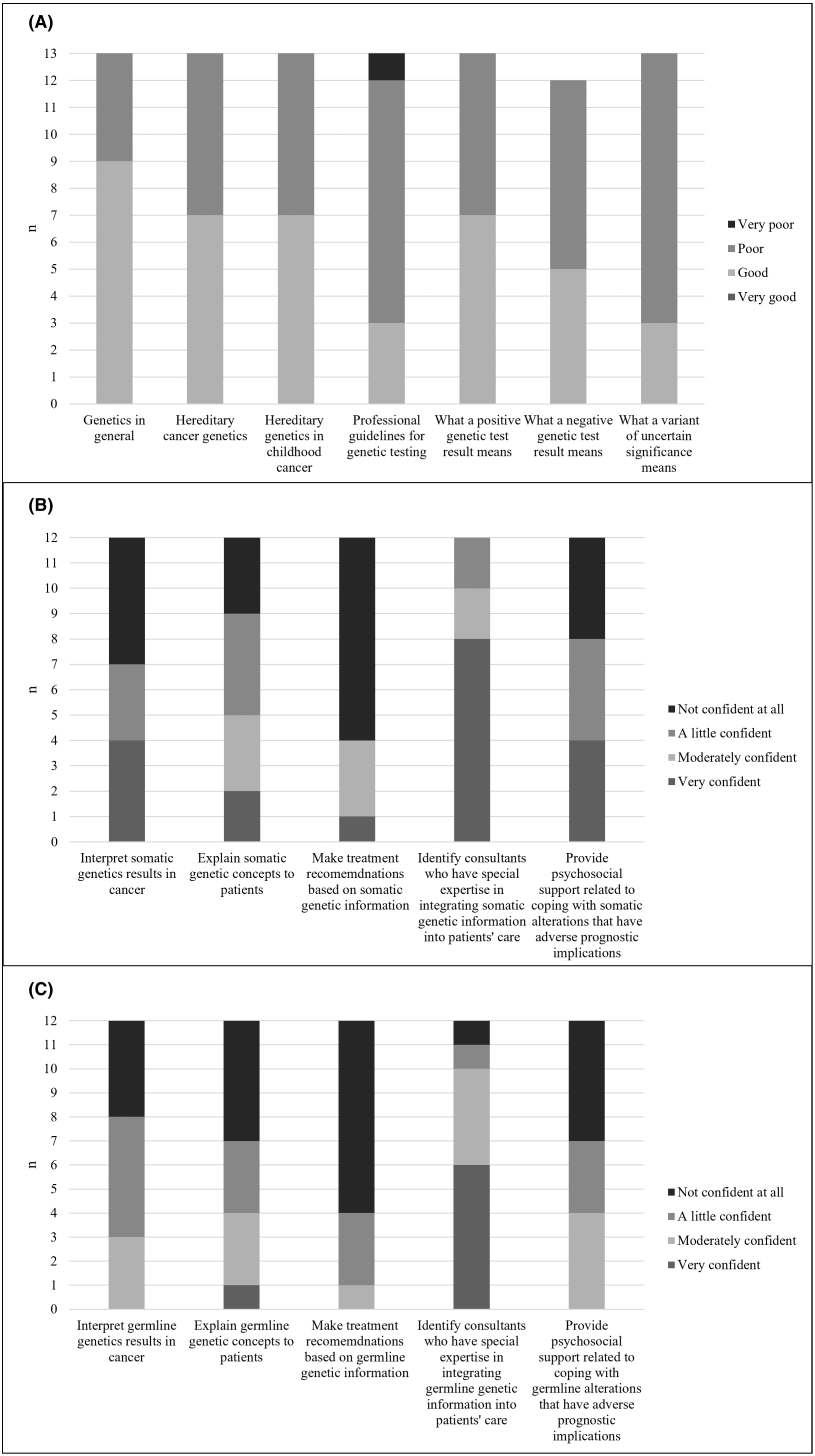
(A) Perceived knowledge of genetic concepts. (B) Confidence of somatic genetic test results. (C) Confidence of germline genetic test results.

Figure 1 presents surgeons' ratings of their perceived knowledge of genetics and their confidence with somatic and germline genetic test results. No surgeon rated their knowledge of genetics across any domain as ‘very good’ (1A) and a minority of surgeons rated themselves as ‘very confident’ across the somatic (1B) and germline (1C) domains other than identifying consultants with special expertise. Qualitatively, surgeons felt their limited knowledge of genetic concepts and specific training could lead to ambiguity in decision‐making. ‘*I don't think I bring anything other than a very rudimentary knowledge to the table*’ (*ID 091*). Some surgeons felt they did not have sufficient knowledge to contribute to the PRISM MTB meetings but expressed an interest in becoming involved to ensure surgical perspectives are represented. Surgeons believed that genetic specialists, whose involvement in patient care was determined on a case‐by‐case basis, should be involved in patient care from trial enrolment, with some describing difficulties in accessing genetic specialists. ‘*Genetic specialists are not always readily at hand, and a referral can take some time*’ (*ID 087*).

## 4 DISCUSSION

We examined the perspectives and experiences of surgeons involved in a precision medicine trial for poor prognosis paediatric cancer patients. Consistent with previous research, surgeons held positive attitudes towards precision medicine.^7^, ^15^ Surgeons were willing to support patient participation by adjusting their surgical schedules to accommodate the need for fresh tissues and were interested in becoming more involved in the PRISM MTB. Nevertheless, some surgeons highlighted ethical concerns performing biopsies solely for research purposes or obtaining larger biopsies for the trial but felt comfortable in advocating against procedures where they believed the risks outweighed the potential benefit to the patient. Like oncologists and scientists,^16^ surgeons expressed concerns that families may hold unrealistic expectations of PRISM.

Surgeons had differing perspectives on many aspects of PRISM, including who they felt benefitted most/least from PRISM so far and the usefulness of obtaining biopsies for research purposes due to uncertainty surrounding potential clinical benefit. Echoing previous research,^17^ surgeons were more willing to proceed with a procedure if the risks to the current patient were low and the benefits high.

Surgeons acknowledged they had limited knowledge and confidence of genetics with no participants rating their knowledge of genetic concepts as ‘very good’. It would be valuable to explore this further in future studies given limited genetic knowledge could contribute to ambiguity in decision‐making. Most surgeons felt very confident in their ability to identify genetic specialists but acknowledged accessing these specialists could be difficult; a challenge recognised in the field as the result of rapidly increasing demand and a limited workforce.^18^

Although our study provides novel and valuable information about surgeons' perspectives of precision medicine, our small sample recruited from a single paediatric precision medicine programme for patients with a poor prognosis represents a limitation that may constrain the interpretation and generalisability of the data. As PRISM is the first precision medicine trial in Australia for children with cancer, our pool of potential participants was restricted.

## 5 CONCLUSION

Surgeons have positive attitudes towards precision medicine but mixed perspectives on how it will benefit their patients. Our findings highlight the need for precision medicine programmes to provide more workforce support to surgeons by establishing collaborative links with genetics teams and facilitating surgeons' inclusion in multidisciplinary meetings. This approach may contribute to the improvement of surgeons' knowledge and confidence in the field. It is important that future work continues to identify and respond to evolving workforce needs as precision medicine expands.

## AUTHOR CONTRIBUTIONS

**Rebecca Daly:** Conceptualization (equal); data curation (lead); formal analysis (lead); investigation (lead); methodology (equal); project administration (lead); validation (lead); visualization (lead); writing – original draft (lead); writing – review and editing (lead). **Kate Hetherington:** Conceptualization (equal); formal analysis (supporting); funding acquisition (equal); methodology (supporting); project administration (equal); resources (lead); supervision (equal); validation (equal); visualization (equal); writing – original draft (supporting); writing – review and editing (equal). **Bethany R. Wadling:** Conceptualization (supporting); formal analysis (equal); methodology (equal); visualization (equal); writing – original draft (supporting); writing – review and editing (supporting). **Chris Jacobs:** Conceptualization (equal); formal analysis (supporting); methodology (supporting); supervision (equal); writing – original draft (supporting); writing – review and editing (supporting). **Jonathan Karpelowsky:** Conceptualization (equal); methodology (supporting); writing – original draft (supporting); writing – review and editing (supporting). **Claire E. Wakefield:** Conceptualization (equal); formal analysis (supporting); funding acquisition (lead); methodology (equal); resources (supporting); supervision (equal); writing – original draft (equal); writing – review and editing (equal).

## FUNDING INFORMATION

RD and KH are supported by the Cancer Institute Translational Program Grant (2021/TPG2112) as well as Luminesce Alliance—Innovation for Children's Health. Luminesce Alliance—Innovation for Children's Health, is a not‐for‐profit cooperative joint venture between the Sydney Children's Hospitals Network, the Children's Medical Research Institute and the Children's Cancer Institute. It has been established with the support of the NSW Government to coordinate and integrate paediatric research. KH is also supported by the Zero Childhood Cancer National Personalised Medicine Program for children with high‐risk cancer, a joint initiative of Children's Cancer Institute and Kids Cancer Centre, Sydney Children's Hospital, Randwick. CW is supported by the NHMRC of Australia (APP2008300). The Behavioural Sciences Unit is proudly supported by the Kids with Cancer Foundation.

## CONFLICT OF INTEREST STATEMENT

The authors declare no conflict of interest.

## Data Availability

Research data are not shared.
